# Generation of marker‐free transgenic hexaploid wheat via an *Agrobacterium*‐mediated co‐transformation strategy in commercial Chinese wheat varieties

**DOI:** 10.1111/pbi.12660

**Published:** 2016-12-20

**Authors:** Ke Wang, Huiyun Liu, Lipu Du, Xingguo Ye

**Affiliations:** ^1^ Institute of Crop Science Chinese Academy of Agricultural Sciences Beijing China

**Keywords:** hexaploid wheat, two independent T‐DNA vectors, co‐transformation, marker‐free transgenic plants, transgene silencing

## Abstract

Genotype specificity is a big problem lagging the development of efficient hexaploid wheat transformation system. Increasingly, the biosecurity of genetically modified organisms is garnering public attention, so the generation of marker‐free transgenic plants is very important to the eventual potential commercial release of transgenic wheat. In this study, 15 commercial Chinese hexaploid wheat varieties were successfully transformed via an *Agrobacterium*‐mediated method*,* with efficiency of up to 37.7%, as confirmed by the use of Quickstix strips, histochemical staining, PCR analysis and Southern blotting. Of particular interest, marker‐free transgenic wheat plants from various commercial Chinese varieties and their F_1_ hybrids were successfully obtained for the first time, with a frequency of 4.3%, using a plasmid harbouring two independent T‐DNA regions. The average co‐integration frequency of the *gus* and the *bar* genes located on the two independent T‐DNA regions was 49.0% in T_0_ plants. We further found that the efficiency of generating marker‐free plants was related to the number of *bar* gene copies integrated in the genome. Marker‐free transgenic wheat plants were identified in the progeny of three transgenic lines that had only one or two *bar* gene copies. Moreover, silencing of the *bar* gene was detected in 30.7% of T_1_ positive plants, but the *gus* gene was never found to be silenced in T_1_ plants. Bisulphite genomic sequencing suggested that DNA methylation in the 35S promoter of the *bar* gene regulatory region might be the main reason for *bar* gene silencing in the transgenic plants.

## Introduction

Hexaploid wheat is an important worldwide food crop that contributes as much as 35% of the calories consumed by the global population (Godfray *et al*., 2010; Shewry, 2009). To meet increasing demand, it is necessary to improve various economically important traits in wheat by genetic engineering approaches (He *et al*., 2011; Tester and Langridge, 2010). Genetic transformation techniques have been used successfully in the improvement of some major crop species, including soya bean, maize and cotton. New varieties of these plants that have been developed by transgenic methods are now planted widely in many countries, offering great benefits to farmers and helping to protect the environment (James, 2016). However, no commercially released genetically modified wheat variety has been developed, owing partially to its much lower transformation efficiency relative to other crops and also to negative public perceptions about transgenic plants (Bhalla, 2006; Harwood, 2012; Li *et al*., 2012). Recently, *Agrobacterium*‐mediated transformation efficiency in wheat has been improved dramatically. The Japan Tobacco Company developed what they call the PureWheat technique for the spring wheat variety Fielder is used (Ishida *et al*., 2015). Employing this PureWheat technique, Richardson *et al*. (2014) successfully transformed two Australian commercial wheat varieties, Westonia and Gladius, with efficiencies of 45% and 32%, respectively. Marker‐free transgenic Fielder and Gladius wheat plants were obtained with an efficiency of 3% without imposing selection pressure during tissue culture step. This dramatic progress will almost certainly promote the development and eventual commercial introduction of transgenic wheat varieties.

The biosafety of commercialized genetically modified plants has attracted considerable public attention. It is considered important to generate marker‐free varieties where the marker gene used for the generation of positive transgenic plants has been eliminated. Marker‐free transgenic plants can be produced by FLP/FRT site‐specific recombination (Nandy and Srivastava, 2011; Nanto and Ebinuma, 2008), Cre/lox site‐specific recombination (Li *et al*., 2007; Mészáros *et al*., 2015; Mlynarova and Nap, 2003; Moravcikova *et al*., 2008), multi‐auto‐transformation (Khan *et al*., 2011), co‐transformation (Miller *et al*., 2002) and using a marker‐free binary vector (McCormac *et al*., 2001). Among these methods, co‐transformation is the most efficient and simple technique (Tuteja *et al*., 2012). Marker‐free transgenic plants have been generated with the use of an *Agrobacterium*‐mediated co‐transformation system with a plasmid that contains two independent T‐DNA regions for many species including tobacco (McCormac *et al*., 2001), soya bean (Xing *et al*., 2000), maize (Miller *et al*., 2002), sorghum (Lu *et al*., 2009), rice (Rao *et al*., 2011) and durum wheat (Wang *et al*., 2016). The average frequencies of co‐transformation of the gene of interest with the selectable marker gene in sorghum and rice were reported to be 45% and 67%, respectively (Lu *et al*., 2009; Rao *et al*., 2011). In addition, marker‐free soya bean plants were obtained at a frequency of 7.2% using an *Agrobacterium*‐mediated co‐transformation plasmid with three independent T‐DNA regions (Ye and Qin, 2008). The establishment of an efficient *Agrobacterium*‐mediated wheat transformation system by the Japan Tobacco Company has laid a solid foundation for routinely obtaining marker‐free transgenic hexaploid wheat plants by employing an *Agrobacterium*‐mediated co‐transformation system.

The objective of this study was to establish a system to produce marker‐free transgenic commercial Chinese hexaploid wheat varieties by *Agrobacterium*‐mediated co‐transformation with two independent T‐DNA regions, and to thereby widen the genotype range for wheat transgenic breeding and variety development. Our study also revealed the silencing reason of selection *bar* gene in the transgenic wheat plants.

## Results

### *Agrobacterium*‐mediated transformation efficiencies of 17 commercial Chinese wheat cultivars

**Figure 1:**
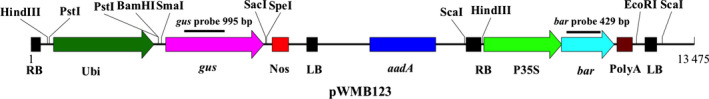
Vector map of pWMB123 showing two T‐DNA regions containing the *bar* and *gus* expression cassettes.

To generate marker‐free transgenic commercial Chinese hexaploid wheat varieties by *Agrobacterium*‐mediated co‐transformation, a universal expression vector pWMB122 for biosafety purpose containing two independent T‐DNA regions was constructed as shown in Figure S1. One T‐DNA region harbours the *bar* selectable marker under the control of the *CaMV35S* promoter and the *Agrobacterium nopaline synthase* (NOS) terminator sequence, and the other T‐DNA harbours the maize ubiquitin (*ubi*) promoter, a multiple cloning site (MCS) and the *NOS* terminator sequence (Figure S1). The *gus* (β‐glucuronidase) reporter gene was inserted into the *BamH*I and *Sac*I sites in the MCS of pWMB122 to form the vector pWMB123 (Figure 1), which was used for all of the transformation experiments in this study.

Immature embryos of 17 commercial Chinese hexaploid wheat varieties, as well as the model wheat line Fielder, were transformed with *Agrobacterium* harbouring the pWMB123 expression vector. After culturing on WLS resting medium, a subset of transformed wheat explants was analysed for transient expression of *gus*. All of the commercial Chinese wheat cultivars tested showed strong *gus* expression in the infected immature embryos (Figure S2), which suggests that the cultivars used in the present investigation were amenable to *Agrobacterium* infection. However, there were large differences among the varieties in the production of primary callus, the production of embryonic callus and shoot regeneration on selection media containing PPT 5–10 mg/L.

**Figure 2:**
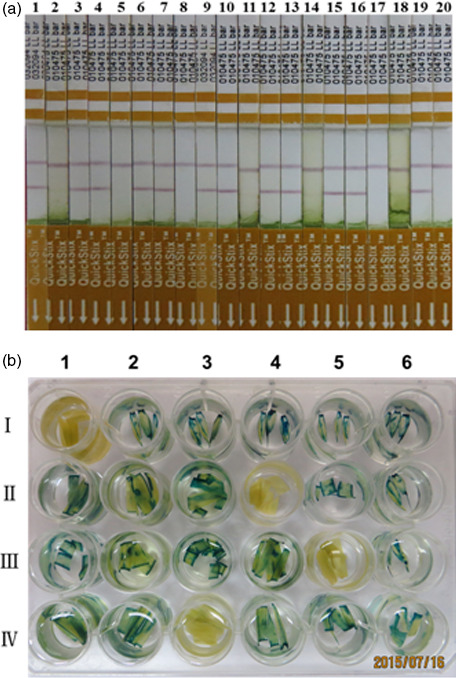
Detection of putative T_0_ transgenic hexaploid wheat plants with QuickStix strips for the *bar* protein (a) and histochemical staining for the *gus* gene (b). a: 1, *bar‐*positive plants from CB037; 2, wild‐type CB037; 3‐4, *bar‐*positive plants from Kenong199; 5, wild‐type Kenong199; 6‐7, *bar‐*positive plants from Xinchun9; 8, wild‐type Xinchun9; 9‐10, *bar‐*positive plants from Jimai5265; 11, wild‐type Jimai5265; 12‐13, *bar‐*positive plants from Shi4185; 14, wild‐type Ahi4185; 15‐16, *bar‐*positive plants from Shiluan02‐1; 17, wild‐type Shiluan02‐1; 18, a *bar‐*negative plant from Jimai22; 19, *bar‐*positive plants from Jimai22; 20, wild‐type Jimai22. b: I1, wild‐type Kenong199; I2‐I6, *gus‐*positive plants from Kenong199; II 1‐ II 3, II 5‐ II 6, *gus‐*positive plants from Xichun9; II 4, wild‐type Xinchun9; III1‐ III 4, III 6, gus‐positive plants from CB037; III 5, wild‐type CB037; IV1‐IV2, IV4‐IV6, gus‐positive plants from Jimai5265; IV3, wild‐type Jimai5265.

**Figure 3:**
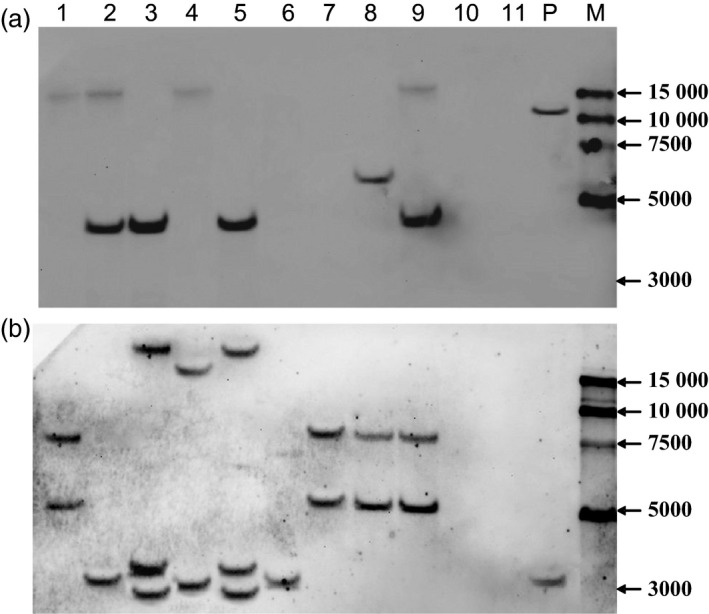
Southern blot analysis of *gus* (a) and *bar* (b) genes copy number in T_0_ transgenic hexaploid wheat plants. 1‐11: T_0_ transgenic plants (1, C1 from CB037, *gus+bar+*; 2, KC2 from the KC hybrid, *gus+bar+*; 3, KC4 from the KC hybrid, *gus+bar+*; 4, KC1 from the KC hybrid, *gus+bar+*; 5, KC3 from the KC hybrid, *gus+bar+*; 6, K1 from Kenong199, *gus−bar+*; 7, X1 from Xinchun9, *gus−bar+*; 8, K3 from Kenong199, *gus+bar+*; 9, C2 from CB037, *gus+bar+*; 10, FC1 from the FC hybrid, *gus−bar−*; 11, Kenong199 as control);P: expression vector pWMB123;M: 15000‐bp DNA marker.

Putative T_0_ transgenic wheat plants were identified based on Quickstix strip detection of *bar* protein (Figure 2a), histochemical analysis of *gus* activity (Figure 2b), and PCR and Southern blot detection of the *bar* and *gus* genes (Figure 3). Transgenic plants were obtained for 15 of the 17 commercial Chinese wheat varieties with transformation efficiencies ranging from 2.7% for Jimai22 to 37.7% for CB037 (Table 1). More than 10% transformation efficiency was achieved for Kenong199, Jimai5265, Zhoumai18, Neimai836, Jingdong18, Xinchun9 and CB037. Of particular note, CB037 reached transformation efficiency close to that of Fielder (45.3%). Only two commercial varieties, AK58 and Jing411, did not yield transgenic plants. These results reveal that most of the commercial Chinese wheat varieties used in this study are able to be transformed by *Agrobacterium*, although most varieties had comparatively low transformation efficiencies compared to Fielder. Among 237 *bar*‐positive T_0_ plants, only 117 plants were confirmed to have the *gus* gene, and the average *gus* and *bar* gene co‐integration rate was 49.0% (Table 1). Southern blotting revealed that the *gus* gene was single copy in most transgenic wheat plants (Figure 3a). However, the *bar* gene integration was observed in a few transgenic plants as a single copy, and in most plants by multiple copies (Figure 3b). This indicates that different numbers of the *gus* gene and the *bar* gene integrated into the same genome. All of the T_0_ positive transgenic plants obtained from the commercial Chinese wheat varieties have normal fertility, and we did not find any sterile transgenic plants.

**Table 1:** *Agrobacterium*‐mediated transformation efficiencies of commercial hexaploid Chinese wheat varieties

Varieties	Growth habit	Number of experiments	Number of Immature embryos transformed	Plants with Liberty activity	Transformation efficiency (%)	Co‐transformation rate (%)
Kenong199	Facultative	3	110	25	22.7	44.0
Shiluan02‐1	Facultative	4	167	16	9.9	38.5
Shi4185	Facultative	3	125	10	8.0	53.3
Chuanmai42	Facultative	4	132	11	8.3	60.0
Jimai5265	Facultative	2	95	14	14.7	51.7
Zhoumai18	Facultative	5	260	35	13.5	58.3
Neimai836	Facultative	4	206	25	12.1	66.7
Zhongmai895	Winter	2	138	5	3.6	NA
Lunxuan987	Winter	2	69	2	2.9	NA
Jingdong18	Winter	3	96	17	17.7	NA
Jimai22	Winter	5	297	8	2.7	NA
AK58	Facultative	6	316	0	0	NA
Zhengmai7698	Facultative	3	188	23	12.2	NA
Yangmai16	Facultative	5	208	12	5.8	NA
Jing411	Winter	4	216	0	0	NA
Xinchun9	Spring	3	165	22	13.3	60.0
CB037	Spring	3	138	52	37.7	50.0
Fielder	Spring	3	181	82	45.3	50.0

NA, not available.

### Transformation efficiencies of F_1_ hybrids derived from crosses between various wheat varieties

Fielder and CB037 (high transformability), Kenong199 and Xinchun9 (medium transformability), and Jimai22 (low transformability) were used to make four hybrid crosses: Kenong199 × CB037 (KC), Kenong199 × Fielder (KF), Xinchun9 × Fielder (XF) and Jimai22 × Fielder (JF). The immature embryos of the four F_1_ hybrids and their parents were transformed with *Agrobacterium* harbouring pWMB123. Transgenes were detected by histochemical staining, Quickstix strips and PCR analysis. Transformation efficiencies of three hybrids, KF, XF and JF, were between the values of their parents, and the transformation efficiency of the hybrid KC was close to the value of its high transformability parent CB037 (Table 2). These results suggest that wheat transformability is a quantitative trait and might therefore be controlled by a few loci. Southern blot analysis was used to detect the integration status of the *gus* and *bar* gene cassettes in a subset of the transgenic plants (Figure 3). It is suggested that the *gus* gene and the *bar* gene were inserted into the genomes of the same transgenic plants by different copy numbers. Also, all of the T_0_ positive transgenic plants obtained from the F_1_ hybrid plants and their parents have normal fertility, and no sterile transgenic plants were found.

**Table 2:** *Agrobacterium*‐mediated transformation efficiencies of four F_1_ hybrids and their hexaploid wheat parents

F_1_ hybrids	Number of experiments	Number of Immature embryos transformed	Plants with *gus* activity	Transformation efficiency (%)
Kenong199	1	54	13	24.1
CB037	1	46	16	34.7
Kenong199 × CB037	2	99	37	37.4
Fielder	2	77	41	53.2
Xinchun9	2	77	10	13.0
Xinchun9 × Fielder	1	55	21	38.2
Kenong199 × Fielder	2	80	30	37.5
Jimai22	2	67	1	1.5
Jimai22 × Fielder	2	78	12	15.4

### Screening for marker‐free transgenic wheat plants and transgene inheritance in the T_1_ generation

**Figure 4:**
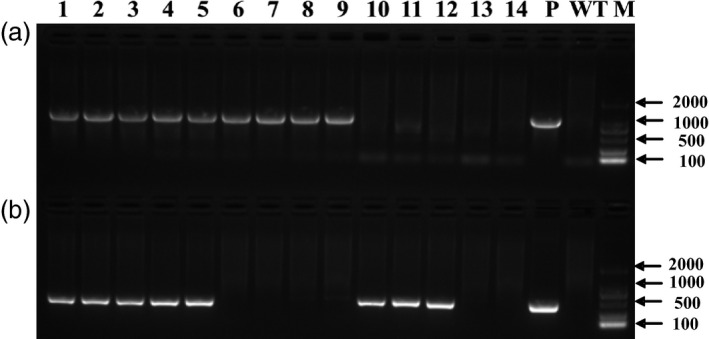
PCR detection of the *gus* (a) and *bar* (b) genes for the screening of marker‐free transgenic plants in the T_1_ generation. 1‐14: individual T_1_ plants (1, X2‐1 from Xinchun9, *gus+bar+*; 2, X2‐3 from Xinchun9, *gus+bar+*; 3, KC2‐1 from the KC hybrid, *gus+bar+*; 4, KC2‐3 from the KC hybrid, *gus+bar+*; 5, J1‐5 from Jimai5265, *gus+bar+*; 6, KC2‐4 from the KC hybrid, *gus+bar−*; 7, KC2‐5 from the KC hybrid, *gus+bar−*; 8, KC2‐6 from the KC hybrid, *gus+bar−*; 9, X2‐4 from Xinchun9, *gus+bar−*; 10, C2‐1 from CB037, *gus−bar+*; 11, K3‐1 from Kenong199, *gus−bar+*; 12, C2‐2 from CB037, *gus−bar+*; 13, FC2‐1 from the FC hybrid, *gus−bar−*; 14, X2‐6 from Xinchun9, *gus*−*bar*−); P: expression vector pWMB123; WT: wild type Xinchun9; M: 2000 bp DNA marker.

**Figure 5:**
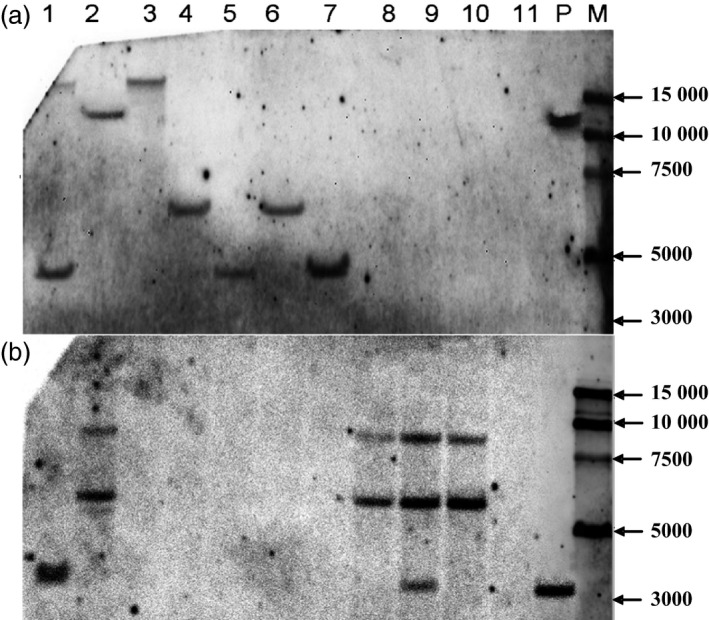
Southern blot detection of *gus* (a) and *bar* (b) genes for the identification of marker‐free transgenic hexaploid wheat plants in the T_1_ generation. 1‐11: different T_1_ individual plants (1, KC2‐1 from the KC hybrid, *gus+bar+*; 2, X2‐1 from Xinchun9, *gus+bar+*; 3, KC2‐2 from the KC hybrid, *gus+bar*−; 4, KC2‐3 from the KC hybrid, *gus+bar* ^*‐*^; 5, KC2‐4 from the KC hybrid, *gus+bar−*; 6, KC2‐5 from the KC hybrid, *gus+bar−*; 7, KC2‐6 from the KC hybrid, *gus+bar*−; 8, C2‐1 from CB037, *gus−bar+*; 9, K3‐1 from Kenong199, *gus−bar+*; 10, C2‐2 from CB037, *gus−bar+*; 11, FC2‐1 from the FC hybrid, *gus−bar−*); P: expression vector pWMB123; M: 15000 bp DNA marker.

To obtain marker‐free transgenic wheat plants based on genotype screening, twelve T_0_ plants were randomly chosen from the 117 plants that tested positive for both the *gus* and *bar* genes, and progeny from these plants were grown in a greenhouse. At the booting stage, every T_1_ plant was tested for the presence and expression of the *gus* and *bar* genes by PCR, *gus* staining and Quickstix strips. Four types of transgenic plants were detected by PCR in the T_1_ generation: *gus+bar+*,* gus+bar−*,* gus−bar+* and *gus−bar−* (Figure 4). Most of the T_1_ plants were *gus+bar+*, whereas there were very few marker‐free plants *gus+bar−* (Table 3). To confirm these results, some plants of each of the four types were selected for Southern blot analysis. According to PCR (Figure 4) and Southern blot analysis (Figure 5), marker‐free plants (*gus+bar−*) were identified in the T_1_ generation of three transgenic lines: KC2 from the KC hybrid, X2 from Xinchun9 and J1 from Jimai5265 with an efficiency of 40.0%, 9.5% and 7.5% (Table 3), respectively.

**Table 3:** Genotyping detection for the *bar* and *gus* genes in the selected T_1_ lines by PCR

T_0_ events	T_1_					*P*‐value	*gus*				*bar*			
*gus+ bar+*	*gus+ bar+*	*gus+ bar‐*	*gus− bar+*	*gus− bar−*	χ^2^ (9:3:3:1)		Presence	Absence	χ^2^ (3:1)	*P*‐value	Presence	Absence	χ^2^ (3:1)	*P*‐value
X2	16	2	2	1	3.47	0.33	18	3	1.29	0.26	18	3	1.29	0.26
X3	14	0	0	3	11.97	0.01	14	3	0.49	0.48	14	3	0.49	0.48
C1	10	0	5	1	4.44	0.22	10	6	1.33	0.25	15	1	3.00	0.08
C2	8	0	6	1	6.45	0.09	8	7	3.76	0.05	14	1	2.69	0.10
C3	12	0	1	2	6.69	0.08	12	3	0.20	0.65	13	2	1.09	0.30
KC1	17	0	6	4	8.62	0.03	17	10	2.08	0.15	23	4	1.49	0.22
KC2	8	6	1	0	5.74	0.12	14	1	2.69	0.10	9	6	1.80	0.18
KC3	21	0	4	4	9.80	0.02	21	8	0.10	0.75	25	4	1.94	0.16
KC4	10	0	6	1	5.69	0.13	10	7	2.37	0.12	16	1	3.31	0.07
KC5	10	0	2	1	3.55	0.31	10	3	0.03	0.87	12	1	2.08	0.15
K3	15	0	10	9	31.57	0	15	19	17.30	0	25	9	0.04	0.84
J1	19	3	15	3	10.84	0.01	22	18	8.53	0.01	34	6	2.13	0.14
Total	160	11	58	30			171	88			218	41		

Transgenic T1 lines X2 and X3 are derived from Xinchun9; C1, C2 and C3 from CB037; KC1, KC2, KC3, KC4 and KC5 from the KC hybrid; K3 from Kenong199; J1 from Jimai5265.

### Analysis of transgene silencing in T_1_ progeny

Based on the screening of twelve T_1_ populations for both the presence of the *gus* and *bar* transgenes and the expression levels of these transgenes, we found that the PCR detection of the *gus* gene corresponded well with the *gus* staining results, but PCR detection of the *bar* gene was inconsistent with Quickstix strip results. Among the 259 T_1_ plants tested, 171 *gus*‐positive plants and 218 *bar*‐positive plants were detected based on PCR (Table 3); 171 plants expressing *gus* were detected by histochemical staining (Table 4), but only 151 plants expressing the *bar* gene were detected by Quickstix strips. It was thus inferred that the *gus* gene is normally expressed in all of the *gus*‐positive T_1_ plants, whereas the *bar* gene was silenced in 30.7% of the *bar*‐positive T_1_ plants. In particular, *bar* gene‐silenced plants accounted for 72.2% and 66.7% of the *bar*‐positive plants from the X2 and C1 T_1_ populations, respectively (Tables 3 and 4).

**Table 4:** Segregation analysis based on the detection of *bar* and *gus* gene expression in T_1_ lines using Quickstix strips and histochemical staining

T_1_ line	Plants tested	*gus*+ bar+	*gus*+ bar−	*gus*− bar+	*gus*− bar−	Chi‐square probability value (bar)	Chi‐square probability value (*gus*)
X2	21	5	13	0	3	3:1 = 0	3:1 = 0.26
X3	17	14	0	0	3	3:1 = 0.48	3:1 = 0.48
C1	16	5	5	0	6	3:1 = 0	3:1 = 0.25
C2	15	5	3	4	3	3:1 = 0.18	3:1 = 0.05
C3	15	12	0	1	2	3:1 = 0.30	3:1 = 0.65
KC1	27	7	10	4	6	3:1 = 0	3:1 = 0.15
KC2	15	8	6	0	1	3:1 = 0.05	3:1 = 0.10
KC3	29	21	0	1	7	3:1 = 0.91	3:1 = 0.75
KC4	17	3	7	5	2	3:1 = 0.008	3:1 = 0.12
KC5	13	9	1	2	1	3:1 = 0.42	3:1 = 0.87
K3	34	11	4	10	9	3:1 = 0.07	3:1 = 0
J1	40	10	12	14	4	3:1 = 0.03	3:1 = 0.003
Total	259	110	61	41	47	NA	NA

Transgenic T1 lines X2 and X3 are derived from Xinchun9; C1, C2 and C3 are derived from CB037; KC1, KC2, KC3, KC4 and KC5 are derived from the KC hybrid; K3 are derived from Kenong199; J1 are derived from Jimai5265.

To understand the mechanism of *bar* gene silencing in the transgenic wheat plants in this study, we first sequenced the *bar* gene in *bar* gene‐silenced plants. We did not observe any sequence changes indicating that silencing is not due to mutation of the *bar* gene. We next evaluated the expression of the *bar* gene in *bar* gene‐silenced plants by RT‐PCR. We could not detect *bar* mRNA (Figure S3), indicating that the *bar* gene is not expressed at the mRNA level in the *bar‐*silenced plants, likely due to transcriptional gene silencing. Thus, we postulated that the *bar* gene silencing in our study might result from DNA methylation.

### Methylation analysis of the 35S promoter region in *bar* gene‐silenced plants

**Figure 6:**
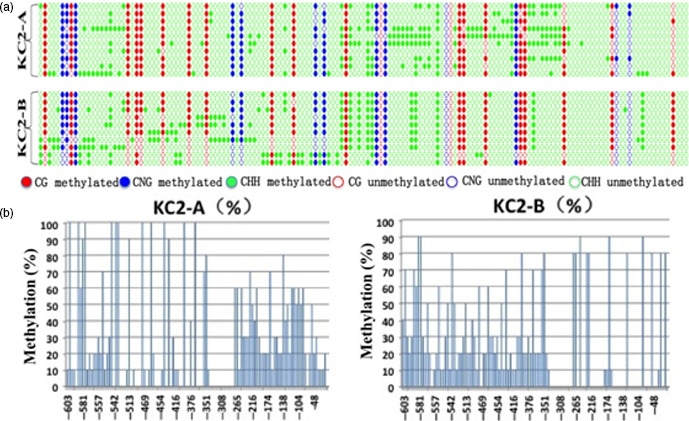
Bisulphite sequencing analysis of the DNA methylation level in the 35S promoter region of *bar*‐expressing and *bar*‐silenced plants. KC2‐A: *bar*‐silenced plants; KC2‐B: *bar*‐expressing plants.

One *bar*‐expressing plant and one *bar*‐silenced plant from the T_2_ generation of the KC2‐1 line were chosen for the evaluation of methylation by bisulphite sequencing. The methylation frequency in the 35S promoter region controlling *bar* gene expression in the *bar*‐silenced plant was obviously higher than that in the *bar*‐expressing plant (Figure 6). The important sites where methylation may result in *bar* silencing are listed in Table S1. There are 11 sites that have a greater than 50% difference in methylation frequency between the *bar*‐silenced and *bar*‐expressing plants (Table S1). Of particular note, the −138 position displayed the largest difference, with a methylation frequency of 80% in the *bar*‐silenced plant and no methylation at all in the *bar*‐expressing plant. The methylation of this site may be a key cause of *bar* gene silencing in the KC2‐1 transgenic wheat plants obtained in this study.

## Discussion

### Transformation ability of commercial Chinese hexaploid wheat cultivars

*Agrobacterium*‐mediated transformation of wheat using immature embryos is known to be strongly genotype dependent. In the past 20 years, transgenic wheat plants were mostly obtained from a few model spring‐type genotypes such as Bobwhite and the Veery (Cheng *et al*., 1997; Khanna and Daggard, 2003). Using the PureWheat technique, another model spring wheat genotype, Fielder, was successfully transformed (Ishida *et al*., 2015). Recently, Richardson *et al*. (2014) achieved *Agrobacterium*‐mediated transformation of eight Australian and Mexican commercial spring wheat varieties, among which Westonia and Gladius showed effective transformation efficiencies. Zhang *et al*. (2015) transformed 10 commercial Chinese wheat varieties by biolistic‐mediated transformation of immature embryos, immature embryo calluses and mature embryo calluses. They obtained transgenic plants from most of the tested wheat varieties, with average efficiencies of 5.15% for immature embryos, 2.26% for immature embryo calluses and 1.89% for mature embryo calluses. Among the 10 varieties tested, three spring or facultative genotypes (Longchun23, Yangmai19 and Kenong199) had transformation efficiencies greater than 7% when immature embryos were used as explants.

In the present investigation, 17 commercial Chinese hexaploid wheat varieties were transformed with *Agrobacterium*, and transgenic wheat plants were generated from 15 of the 17 varieties. One winter cultivar, Jingdong18; two spring cultivars, CB037 and Xingchun9; and six facultative cultivars, including Kenong199, Jimai5265, Zhoumai18, Neimai836, Zhengmai7698 and Yangmai16, were found to have good transformability by *Agrobacterium* (Table 1). Combined with the previous results of Zhang *et al*. (2015), it seems clear that most commercial Chinese wheat varieties can be successfully transformed by *Agrobacterium* or bombardment approaches, and some varieties (such as CB037, Xingchun9, Kenong199, Jimai5265 and Yangmai19) can be potentially used in wheat transgenic breeding or functional genomics studies, owing to their high transformation efficiencies. It should be noted that spring and facultative wheat varieties generally display higher transformation efficiencies than winter wheat varieties. The establishment of an efficient *Agrobacterium*‐mediated wheat transformation system using immature embryos will enable genome editing of genes controlling important traits by techniques such as CRISPR/Cas9 (Wang *et al*., 2014; Ye, 2015).

### Overcoming transformation recalcitrance in commercial wheat varieties

In this investigation, an efficient *Agrobacterium*‐mediated transformation protocol was developed for 15 commercial Chinese wheat varieties, but some varieties remain difficult to transform; these varieties include Jimai22, Jing411 and AK58. We found that the transformation efficiency for recalcitrant commercial wheat varieties could be improved by crossing them with the more‐easily‐transformed varieties. Four F_1_ wheat hybrids derived from crosses between varieties with different transformation efficiencies had elevated transformation efficiencies compared to their recalcitrant parents (Table 2). Application of F_1_ hybrids could alleviate the difficulty in transforming recalcitrant commercial wheat varieties. This strategy, which involves first obtaining transgenic plants from F_1_ hybrids between model genotypes with high transformation efficiencies and commercial wheat varieties with low transformation efficiencies, and then backcrossing the transgenic plants with the commercial varieties for several generations while using molecular markers to select for the target transgenes, might be more efficient than traditionally used breeding methods, which includes first obtaining stable transgenic lines using model genotypes with high transformation efficiencies, and then crossing these lines followed several generations backcrossing with commercial varieties in combination with the tracing of the target transgenes.

### Efficient generation of marker‐free transgenic hexaploid wheat plants

To date, transgenic wheat has not been approved for commercial release, although the development of transgenic wheat plants with various trait enhancements has been reported extensively (Harwood, 2012; Li *et al*., 2012). Several transgenic wheat lines have been evaluated in the field for herbicide resistance (Zhou *et al*., 2003), disease resistance (Chen *et al*., 2014), yield (Chen *et al*., 2014; Zhou *et al*., 2003), gene flow (Dong *et al*., 2016; Rieben *et al*., 2011), interactions between transgenes and the environment (Dong *et al*., 2016; Zeller *et al*., 2010) and effects on microbial community diversity and the impact of enzyme activity on soil (Wu *et al*., 2014). It seems likely that the elimination of selection markers and unnecessary vector backbone DNA in early generations will be helpful in overcoming biosafety limitations and in allowing environmental release of genetically modified wheat (Wang *et al*., 2016).

Marker‐free transgenic wheat plants have been obtained using minimal gene expression cassettes and a cold‐inducible Cre/lox system introduced by biolistic particles (Mészáros *et al*., 2015; Yao *et al*., 2007), as well as via *Agrobacterium‐*mediated methods that employ no selection regime (Richardson *et al*., 2014). Using a co‐transformation technique mediated by *Agrobacterium*, a higher frequency of marker‐free plants was acquired in model plants like tobacco and rice (McCormac *et al*., 2001; Rao *et al*., 2011). However, although *Agrobacterium*‐mediated co‐transformation of two T‐DNAs is known as a reliable and efficient strategy to obtain marker‐free transgenic plants, use of this method has only been reported durum wheat (Wang *et al*., 2016) and not yet in hexaploid wheat. In this study on the generation of marker‐free hexaploid wheat transgenic plants by co‐transformation, the average co‐integration frequency of the *gus* and *bar* genes in T_0_ plants was 49.0% (ranging from 38.5% to 66.7%), which is consistent with the results obtained for other plant species (Lu *et al*., 2009; Rao *et al*., 2011). Eleven transgenic wheat plants with the target gene and without the selection marker (*gus+bar−*) were screened from three T_1_ populations, KC2, X2 and J1. Among 12 T_1_ populations or 259 T_1_ plants in total, the average frequency of obtaining marker‐free lines was 4.3% (Table 3). In the three transgenic T_1_ lines, KC2, X2 and J1, the frequency of marker‐free plants obtained was 40%, 14.3% and 7.5%, respectively. Notably, there was only one to two copies of the *bar* gene in these T_1_ lines. However, marker‐free plants were not obtained from T_1_ plants with multiple copies of the *bar* gene. Therefore, we speculate that the integration copy number of the *bar* gene is perhaps closely related to the efficiency of obtaining marker‐free plants. It appears that marker‐free transgenic plants are relatively easily obtained only for the offspring of T_0_ plants with a single or a few copies of the *bar* gene. Potential reasons for why we were unsuccessful in obtaining *gus+bar−* plants for the other nine T_1_ populations include: (1) the T_1_ population for each line was not large enough and (2) there were more than three copies of the *bar* gene integrated into the genomes of these nine T_0_ plants. Typically, transgenic plants with a single gene copy are selected for functional analysis. In *Agrobacterium*‐mediated co‐transformation experiments, the transgenic plants with a single copy of the selection gene and a single copy of target gene are ideal materials to produce marker‐free plants in the segregation generations at a theoretical rate of 18.75%. For this purpose, the number of copies of the selection marker and of the target gene should be determined in plants of the T_0_ generation.

In the expression vector pWMB123 used in this study, the T‐DNA region containing the *bar* gene was 1.7 kb in length, but the other T‐DNA region containing the *gus* gene was 4.4 kb in length, which is more than two times greater (Figure 1). The unmatched size of the two T‐DNAs may have led to more copies of the *bar* gene than the *gus* gene being integrated into the wheat genome (Figure 3). A previous study reported that different size of the two T‐DNA cassettes in a vector resulted in their different integration copy numbers in transgenic tobacco (McCormac *et al*., 2001). Based on our results, we supposed that the shorter *bar* gene cassette has a higher chance of being inherited via independent assortment in the T_1_ generation. Therefore, decreased *bar* gene integration could be achieved by changing the sizes of the two independent T‐DNA regions in the binary vector to an optimal ratio, by which the rate of successful generation of marker‐free plants would be further improved.

### Possible ways to reduce transgene silencing

In a previous study, marker‐free soya bean plants were obtained at a low frequency using an expression vector containing three independent T‐DNAs including two 3.7 kb target gene cassettes and one 2.5 kb *bar* gene cassette; the *bar* gene was found to be silenced in many positive T_1_ plants (Ye and Qin, 2008). In the present study, among the 218 *bar* gene‐positive T_1_ plants, 67 plants exhibited *bar* gene silencing (30.7%). However, the *gus* gene was not silenced in any of the positive T_1_ plants (Tables 3 and 4).

Gene silencing typically acts in a regional, rather than in a promoter‐ or sequence‐specific manner and generates large domains of DNA that are inaccessible to DNA binding proteins. Anandalakshmi *et al*. (1998) reported two major mechanistic classes for gene silencing: transcriptional gene silencing (TGS) and post‐transcriptional gene silencing (PTGS). In our *bar* gene‐silenced transgenic wheat plants, mRNA expression analysis did not detect the expression of the *bar* gene. We hypothesize that DNA methylation may be responsible for the *bar* gene silencing. In fact, gene silencing in transgenic plants caused by methylation in the 35S promoter region has been described recently (Okumura *et al*., 2016; Yamasaki *et al*., 2011). In the present experiment, bisulphite sequencing was used to reveal the reasons for *bar* gene silencing. The results indicated that *bar* gene silencing in our transgenic wheat plants was in fact due to DNA methylation of the 35S promoter region, especially the methylation of the cytosine at −138 position. One reason might be that the 35S promoter is easily methylated. In a recently published study, no silencing of the *bar* gene expressed under the control of the ubiquitin promoter in transgenic durum wheat plants was reported (Wang *et al*., 2016). Hence, to alleviate gene silencing in transgenic hexaploid wheat and to efficiently generate marker‐free plants in the T_1_ generation, we propose using other strong promoters (such as ubiquitin and actin) and adjusting the size of the T‐DNA region harbouring the selection gene to reduce the number of copies of the selection gene integrated into the genome.

## Experimental procedures

### Plant materials

Hexaploid wheat genotypes used in this study included spring varieties, including CB037, Xinchun9 and Fielder, and facultative or winter varieties, including AK58, Jimai22, Neimai836, Zhengmai7698, Chuanmai42, Zhoumai18, Shiluan02‐1. Kenong199, Shi4185, Jimai5265, Yangmai16, Lunxuan987, Jing411, Jingdong18 and Zhongmai895. CB037 was developed and kindly provided by Prof. Xiao Chen from Institute of Crop Science, Chinese Academy of Agricultural Science (ICS‐CAAS). Zhongmai895 was developed and kindly provided by Prof. Binghua Liu from ICS‐CAAS. Zhongmai895 was developed and kindly provided by Dr. Yong Zhang from ICS‐CAAS. Jimai5265 was developed and kindly provided by Prof. Hui Li at Institute of Cereal and Oil Crops of the Hebei Academy of Agriculture and Forestry Sciences. The remaining wheat cultivars were acquired from the National Crop Germplasm Bank of ICS‐CAAS. Jimai22 is the number one wheat variety in China and is planted on 2 million hectares each year. Yangmai16 is the number one variety in southern China and is planted on 0.3 million hectares each year. The four F_1_ hybrids, Kenong199 × CB037 (KC), Kenong199 × Fielder (KF), Xinchun9 × Fielder (XF) and Jimai22 × Fielder (JF), were developed previously by our research group.

The spring wheat varieties and the four F_1_ hybrids were directly sown in growth chambers. Winter wheat varieties were germinated in culture dishes (90 mm × 20 mm) at room temperature for 2 days and maintained in a cooler at 4 °C for 30 days for vernalization before sowing. All wheat cultivars were grown in pots (20 cm × 30 cm) filled with substrate peat moss (King Root, Latvia), mixed with patterned released fertilizer (Osmocote Extract, Germany) and maintained with an environmental growth condition regime of 24 °C, 16 h light/18 °C, 8 h dark with 300 μmol/m^2^/s light intensity at 45% humidity. During the growth period, water was supplied once a week. Aphids were controlled with sticky coloured cards (Zhengzhou Oukeqi Instruments Ltd., China); powdery mildew was controlled by application of triadimefon (Jinan Luba Pesticides Co., China).

### Construction of an expression vector with two independent T‐DNA regions

The pZP201 and pPTN290 plasmids were kindly provided by Dr. Tom Clemente of the University of Nebraska‐Lincoln. Plasmids pWMB006 and pWMB010 were constructed previously in our laboratory. The construction of an expression vector with two independent T‐DNA regions was performed as follows: the pWMB006 and pZP201 plasmids were double digested with *Bam*HI and *Eco*RI. The 806‐bp and 7121‐bp fragments were selected and then ligated together to form an intermediate vector, pZP201‐NOS. pZP201‐NOS and pWMB010 were digested with *Sca*I to release 7827‐bp and 3398‐bp fragments, respectively. These two fragments were ligated to form another intermediate vector, pZP201‐NOS‐bar. Next, pZP201‐NOS‐bar and pWMB006 were digested with *Pst*I to release 11225‐bp and 1988‐bp fragments, respectively. The expression vector pWMB122 harbouring two independent T‐DNA regions was constructed by ligating these two fragments (Figure S1). Finally, the expression vector pWMB123 was constructed by ligating the 2077‐bp PCR product amplified from pPTN290 with the primers *gus‐Bam*HI‐F: 5′‐AAAGGATCCATGGTAGATCTGAGGGTA‐3′ and *gus‐Sac*I‐R: 5′‐AAAGAGCTCTCACCTGTAATTCACACGTGG‐3′ into pWMB122, which was double digested with *BamH*I and *Sac*I.

### Bacterial strains and triparental mating

*Agrobacterium* strain C58C1 and an *E. coli* strain containing the helper plasmid pRK2013 were kindly provided by Dr. Tom Clemente, of the University of Nebraska‐Lincoln. The pWMB123 expression vector was introduced into *Agrobacterium* strain C58C1 by triparental mating (Ditta *et al*., 1980).

### *Agrobacterium*‐mediated transformation of wheat immature embryos

Wheat heads from plants grown in a growth chamber were tagged at anthesis and harvested 14 days postanthesis (DPA). In aseptic conditions, immature wheat grains were carefully collected, then surface sterilized with 70% ethanol for 1 min, with 5% sodium hypochlorite (NaClO) for 15 min, and finally rinsed 5 times with sterile water. We used a proprietary method for *Agrobacterium*‐mediated transformation of wheat developed by Japan Tobacco Company (Ishida *et al*., 2015) with slight modifications. In brief, immature wheat embryos were carefully taken from the sterilized grains aseptically under a stereoscopic microscope, incubated with *Agrobacterium* strain C58C1 harbouring pWMB123 for 5 min in WLS‐inf medium at room temperature, and co‐cultivated for 2 d on WLS‐AS medium, with the scutellum facing upwards, at 25 °C in darkness. After co‐cultivation, embryonic axes were removed with a scalpel and the scutella were transferred onto plates containing WLS‐Res medium. After 5 day, the tissues were transferred onto callus induction (WLS‐P5) medium. After 2 weeks, callus cultures were sliced vertically into halves and evenly placed on WLS‐P10 medium for 3 weeks in darkness. Embryogenic calli were then differentiated on LSZ‐P5 medium at 25 °C with 100 μmols/m^2^/s light. Regenerated shoots were transferred into cups filled with MSF‐P5 medium for elongation and root formation. Plantlets with well‐developed root systems were transplanted into pots and cultivated in growth chambers at 25 °C with 300 μmol/m^2^/s light.

### Histochemical staining and Quickstix detection of transgenic wheat plants

Histochemical staining of *gus* expression was conducted as described by Jefferson *et al*. (1987). The transformed immature wheat embryos just after resting culture on WLS‐Res medium or the young leaves of putative transgenic wheat plants were immersed directly in X‐gluc staining buffer (0.1 m NaPO_4_ buffer pH 7.0, Na_2_‐EDTA 10 mm, ferricyanide 0.2 mm, ferrocyanide 0.2 mm, X‐gluc 0.8 g/L, methanol 20%, Triton X‐100 0.5%) and incubated overnight at 37 °C.

Putative transgenic wheat plants and their self‐pollinated offspring were identified based detection of the *bar* protein with the QuickStix Kit (EnviroLogix) for LibertyLink (the expressed protein of the *bar* selection marker gene), according to the manufacturer's instructions.

### PCR‐based genotyping and Southern blot analysis of transgenic wheat plants

Genomic DNA was extracted from the leaves of the T_0_ and T_1_ transgenic plants using a NuClean PlantGen DNA kit (CWBIO, China). The presence of the *gus* gene in the transgenic plants was demonstrated by PCR amplification of a 995‐bp fragment using the primer pair 5′‐CAAGGAAATCCGCAACCATATC‐3′ and 5′‐TCAAACGTCCGAATCTTCTCCC‐3′. The presence of the *bar* gene in the transgenic plant samples was demonstrated by PCR amplification of a 429‐bp fragment using the primer pair 5′‐ACCATCGTCAACCACTACATCG‐3′ and 5′‐GCTGCCAGAAACCACGTCATG‐3′.

For Southern blotting analysis of transgenic wheat plants, total genomic DNA was extracted by employing a standard CTAB method (Sambrook *et al*., 1989) to obtain a larger amount of DNA; 10 μg DNA from each sample was digested with *Hin*dШ because there is only one cutting site for this enzyme adjacent to the RB sequence of the T‐DNA cassettes for the *gus* gene and the *bar* gene on the expression vector pWMB123 (Figure 1). The digested DNA samples were fractionated on a 0.8% agarose gel and transferred onto a nylon Hybond‐N^+^ membrane (Roche, Germany) with a membrane transfer instrument (Model 785, Bio‐Rad). The *gus* (995‐bp) and *bar* (429‐bp) PCR products were labelled with Digoxigenin and used as probes to hybridize with the digested DNA on the membrane. The hybridization and detection steps were performed according to the instructions for the DIG High Prime DNA Labeling and Detection Starter Kit II (Roche, Germany).

### Bisulphite sequencing

Genomic DNA was isolated from transgenic wheat seedlings using a NuClean PlantGen DNA Kit (CWBIO, China). Bisulphite treatment of genomic DNA was conducted using a Methylation Gold Kit according to the manufacturer's instructions (ZYMO Research Corporation). The bisulphite‐treated DNA was then subjected to PCR amplification using Hot‐Start Taq DNA polymerase (R110A, TaKaRa), using the primers listed in Table S2. Sequenced data were analysed using KISMETH (Gruntman *et al*., 2008). At least 10 independent clones from each PCR product were sequenced.

## Conflict of interest

The authors declare no conflict of interests.

## Supporting information


**Figure S1.** Construction of pWMB122, which contains two independent T‐DNA regions.
**Figure S2.** Transient *gus* expression in the immature embryos of various commercial Chinese hexaploid wheat varieties after 5 days of co‐cultivation with *Agrobacterium* harboring pWMB123.
**Figure S3.** RT‐PCR analysis of *bar* gene expression in the KC2‐1 transgenic line. Lanes 1‐7: The *bar*‐expressed plants tested by Quickstix strips; Lanes 8‐14: The *bar*‐silenced plants tested by Quickstix strips; Lane 15: Plasmid pWMB123; Lane 16: Kenong199; Lane17: DL2000 marker.
**Table S1.** Comparison of methylation frequencies at candidate methylation sites in the *bar* gene‐silenced plant KC2‐A and the *bar* gene‐expressing plant KC2‐B.
**Table S2.** Primers used for DNA methylation analysis.
