# Supplementary material for: Generation of marker‐free transgenic hexaploid wheat via an Agrobacterium‐mediated co‐transformation strategy in commercial Chinese wheat varieties
Source: Plant Biotechnol J. 2016 Dec 20;15(5):614–23. doi: 10.1111/pbi.12660 (PMC5399001; doi:10.1111/pbi.12660)
Supplement: Supplementary file 1 — Figure S1. Construction of pWMB122, which contains two independent T‐DNA regions. Figure S2. Transient gus expression in the immature embryos of various commercial Chinese hexaploid wheat varieties after 5 days of co‐cultivation with Agrobacterium harboring pWMB123. Figure S3. RT‐PCR analysis of bar gene expression in the KC2‐1 transgenic line. Lanes 1‐7: The bar‐expressed plants tested by Quickstix strips; Lanes 8‐14: The bar‐silenced plants tested by Quickstix strips; Lane 15: Plasmid pWMB123; Lane 16: Kenong199; Lane17: DL2000 marker. Table S1. Comparison of methylation frequencies at candidate methylation sites in the bar gene‐silenced plant KC2‐A and the bar gene‐expressing plant KC2‐B. Table S2. Primers used for DNA methylation analysis. [file PBI-15-614-s001.doc]

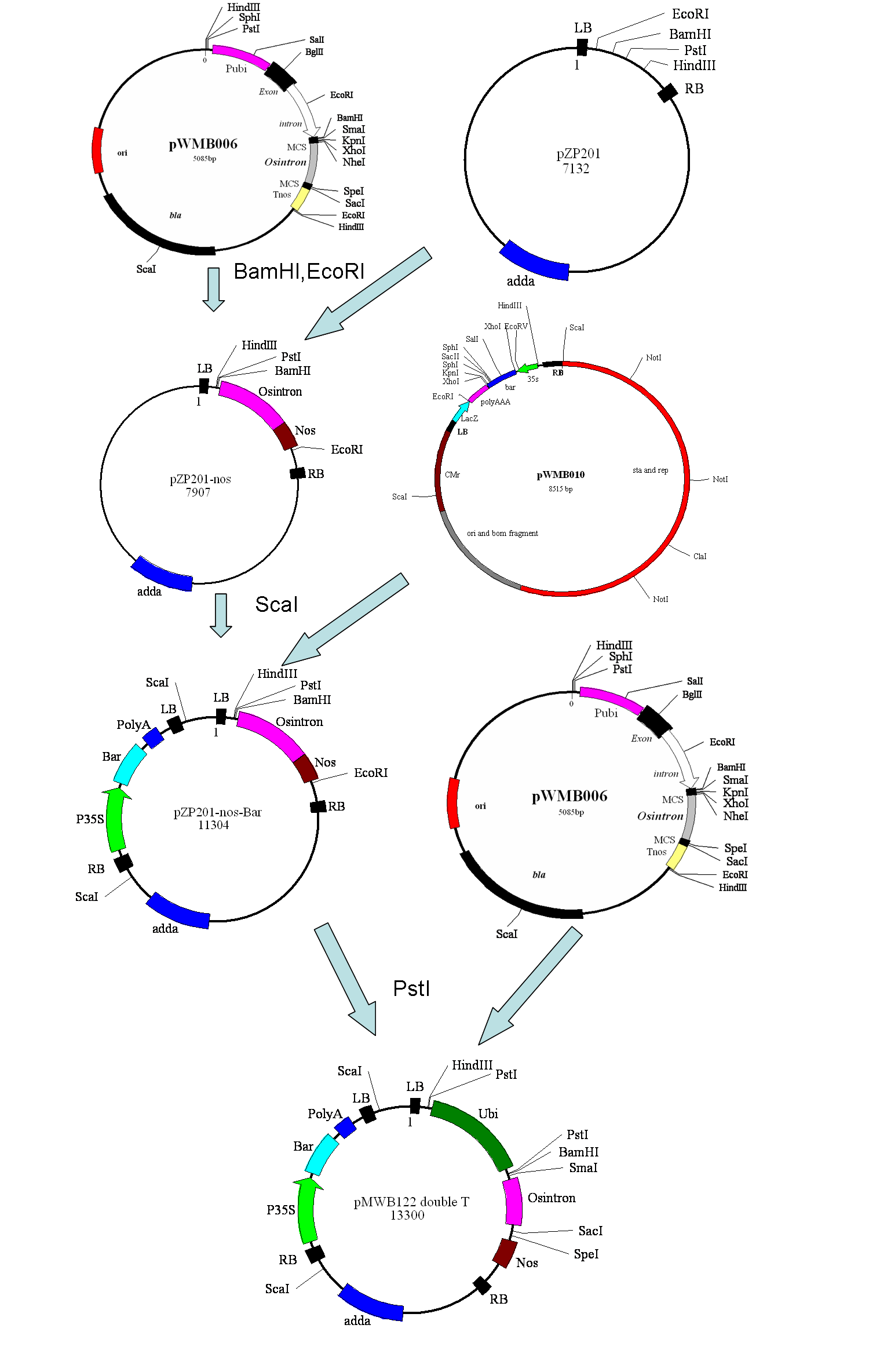


**Figure S1.** Construction of pWMB122, which contains two independent T-DNA regions.


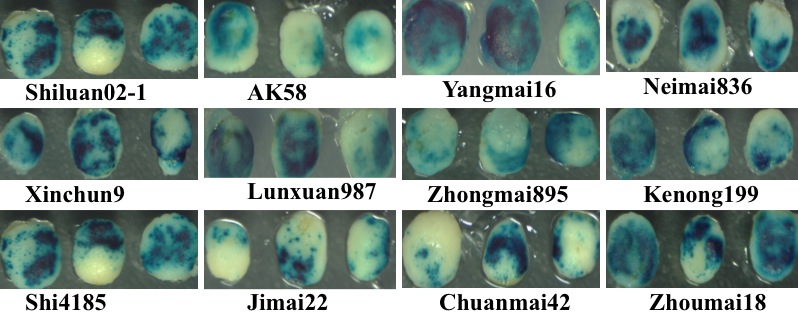


**Figure S2.** Transient *gus* expression in the immature embryos of various commercial Chinese hexaploid wheat varieties after 5 days of co-cultivation with *Agrobacterium* harboring pWMB123.


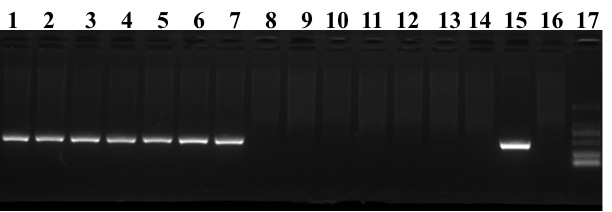


**Figure S3.** RT-PCR analysis of *bar* gene expression in the positive KC2-1 transgenic line. Lanes 1-7: The *bar*-expressed plants tested by Quickstix strips; Lanes 8-14: The *bar*-silenced plants tested by Quickstix strips; Lane 15: Plasmid pWMB123; Lane 16: Kenong199; Lane17: DL2000 marker.

**Table S1.** Comparison of methylation frequencies at the candidate methylation sites in the *bar* gene-silenced plant KC2-A and the *bar* gene-expressing plant KC2-B.

| Position | KC2-A（%） | KC2-B（%） |
| --- | --- | --- |
| -549 | 100 | 50 |
| -524 | 100 | 50 |
| -437 | 100 | 50 |
| -216 | 50 | 0 |
| -214 | 60 | 0 |
| -138 | 80 | 0 |
| -111 | 60 | 0 |
| -110 | 60 | 0 |
| -108 | 50 | 0 |
| -104 | 60 | 0 |
| -103 | 50 | 0 |

**Table S2.** Primers used in the present study for DNA methylation analysis.

| Name | Sequences |
| --- | --- |
| 1F | ATTGCGATAAAGGAAAGG |
| 1R | CCTTCCTTTTCTACTATCCTTTTAA |
| 2F | GTTTTTGGAGGTATAGGGTTTTAAGA |
| 2R | AATCCAACTACCAAAAACCCAC |
| 6F | TTTAGATAAGGGAATTAGGG |
| 6R | TACCGACAAACTAAAATCCAACTAC |
| ZKF2 | CGACGGTTAGTGTTAAGTTT |
| ZR2 | ATACAAACCGCCGACATA |
